# Stiff auxetics: Hierarchy as a route to stiff, strong lattice based auxetic meta-materials

**DOI:** 10.1038/s41598-018-30822-x

**Published:** 2018-08-20

**Authors:** D. Rayneau-Kirkhope

**Affiliations:** Department of Applied Physics, Aalto University, 02150 Espoo, Finland

## Abstract

Using a combination of analytic and computational methods, we examine the effect of adding hierarchical substructure to an auxetic lattice. Our novel methodology, involving a coarse grain approach, allows for the analysis of hierarchically sub-structured lattices where direct computation would prove intractable. We show that through hierarchy one can create ultra-lightweight auxetic meta-materials of high strength and stiffness. Through scaling law arguments, we show that the benefits of hierarchical design can also be obtained in the general class of bending-dominated lattices. Furthermore, we show that the hierarchical structures presented show a wide range of tailorability in their mechanical properties, and exhibit increased strength when optimised for buckling resistance. Auxetic materials have a broad range of potential applications, and thus the creation of ultra-light auxetic meta-materials with enhanced stiffness and strength is undoubtedly of practical importance.

## Introduction

When a material is compressed our intuition tells us that it should expand in a direction perpendicular to the applied load. Poisson’s ratio is a fundamental material property that quantifies this phenomenon: It is defined as the negative ratio of transverse strain to the imposed longitudinal strain. The Poisson’s ratio of a material is therefore positive when it behaves in accordance with our intuition. When a material deviates from this behavour (that is to say, it has a negative Poisson’s ratio) it is termed auxetic^1^. Auxetic materials are of broad interest across the sciences^2–6^ due to their wide range of potential applications including novel fasteners^7^, biomedical use^8^, particle filters^9^, scaffolds for reconfigurable metasurfaces^10^, and energy damping/transmitting devices^11^.

Natural materials with negative Poisson’s ratio are rare^12^, however, owing to their potential applications in many areas of technology, numerous mechanisms have been designed to induce auxetic behaviour in mechanical meta-materials. These mechanisms include rotating elements^13–17^, lattice based geometries^8,18,19^, rigid link structures^20,21^, and origami (paper folding)^22–24^ and kirigami (paper cutting)^25–27^ based designs (for an overview of such mechanisms, see the reviews^28,29^ among others). Elastic instability has also been used to induce pattern formation that results in auxetic meta-material behaviour^30–32^. In this article, we will focus on the behaviour of lattice based, auxetic meta-materials.

The mechanics of lattice based materials can be broadly split into two categories: bending-dominated and stretching-dominated^33^. These terms describe the dominant deformation mode when the material is subject to an external stress - the members bend or stretch to accommodate an imposed displacement. Lattices with low connectivity tend to be bending dominated, while those with higher connectivity tend to be stretching-dominated^34^. These two regimes are seen to have markedly different global mechanical properties^33,34^. For example, the two classes of lattice have distinct scaling laws relating the relative stiffness, $\tilde{E}/E$, and the relative density, $\tilde{\rho }/\rho$, of the lattice based materials; these scaling laws are given by^33^

1 $$\frac{\tilde{E}}{E}\sim {(\frac{\tilde{\rho }}{\rho })}^{\alpha }$$

where *α* = 1 for stretching-dominated lattices and *α* = 3 for bending-dominated lattices in 2-d. In the above expression, *E* and $\tilde{E}$ are the Young’s modulus of the construction material and lattice based meta-material respectively, while *ρ* and $\tilde{\rho }$ are the density of the construction material and the meta-material respectively. Knowing these scaling relationships, it is clear that for lightweight materials ($\tilde{\rho }/\rho \ll 1$), stretching-dominated lattices have higher stiffness. In all auxetic materials constructed from a lattice of uniform beams, restricted to in-plane deformations, the underlying mechanics of the system has been observed to be bending-dominated, and it has been conjectured that this must be so in general^35^ and that this would imply that there exisits a fundamental limit to the stiffness of lightweight auxetic materials^35^. However, as demonstrated in this paper, one can utilise hierarchy to design a lattice where at the longest length-scale the deformation mode appears to be bending-dominated, while retaining the beneficial scaling relationship relating stiffness to relative density typically associated with stretching-dominated architectures. In utilising structural hierarchy to create structures combining multiple advantageous mechanical properties, we follow a trend often observed in nature^36,37^: structural hierarchy is observed in many naturally occurring structures and that hierarchy is closely associated with remarkable mechanical properties. The combination of elasticity and strength of spider silk^38^, stiffness and toughness of nacre^39,40^, and low weight and high stiffness of trabecular bone^41^ are all properties created through hierarchical geometries.

In order to demonstrate how substructure can be used to create ultralightweight, stiff auxetic materials, we take the motif of a well studied bending dominated architecture^18^ and utilise a specific sub-lattice architecture in order to ensure that the analogous freely-hinged structure exhibits no mechanism. Such a lattice has been termed a “bending-stretching” dominated lattice^42,43^. Through this combination, we obtain an auxetic lattice that has superior stiffness to weight ratios than those without substructure. We then iterate this procedure in order to create auxetic lattices with an arbitrary degree of hierarchy. We present a scaling law argument showing that lightweight auxetic lattices of unprecedented stiffness (*α* = 1 in Eq. (1)) can be created through this methodology. This prediction is then confirmed by full finite element simulation on the hierarchical lattice structure. It is noted that this use of slender frame elements results in a scaling relationship that exceeds that predicted in previous work on hierarchical lattices^43^, leading to stiffer structures in the limit of lightweight materials. Furthermore, through the addition of hierarchy, we show that the scaling relationship of buckling load (and therefore plateau stress in the stress-strain curve^33^), can be manipulated leading to a design that has an improved stiffness and strength to weight ratios compared with lattices made up of simple beam elements. It is notable that in contrast to other works that focus on stiffening individual lattice architectures^44,45^, the methodology presented here, and the analytically derived scaling laws for both strength and stiffness, are applicable to the broad range of bending dominated lattices. To highlight this, two further bending dominated lattices are considered, one auxetic and one non-auxetic, both showing the same scaling relationships.

## Geometry

**Figure 1:**
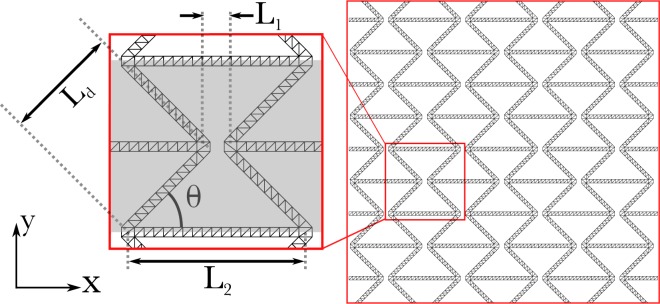
Globally auxetic lattice with minimally rigid sub-lattice: Such a structure allows the design of auxetic response while retaining efficiency of a stretching dominated lattice structure. The figure also introduces the notation used to parameterise the geometry of the lattice. The grey box (inset) shows one unit cell, the lattice investigated here is made up of *N*_*x*_ by *N*_*y*_ unit cells.

**Figure 2:**
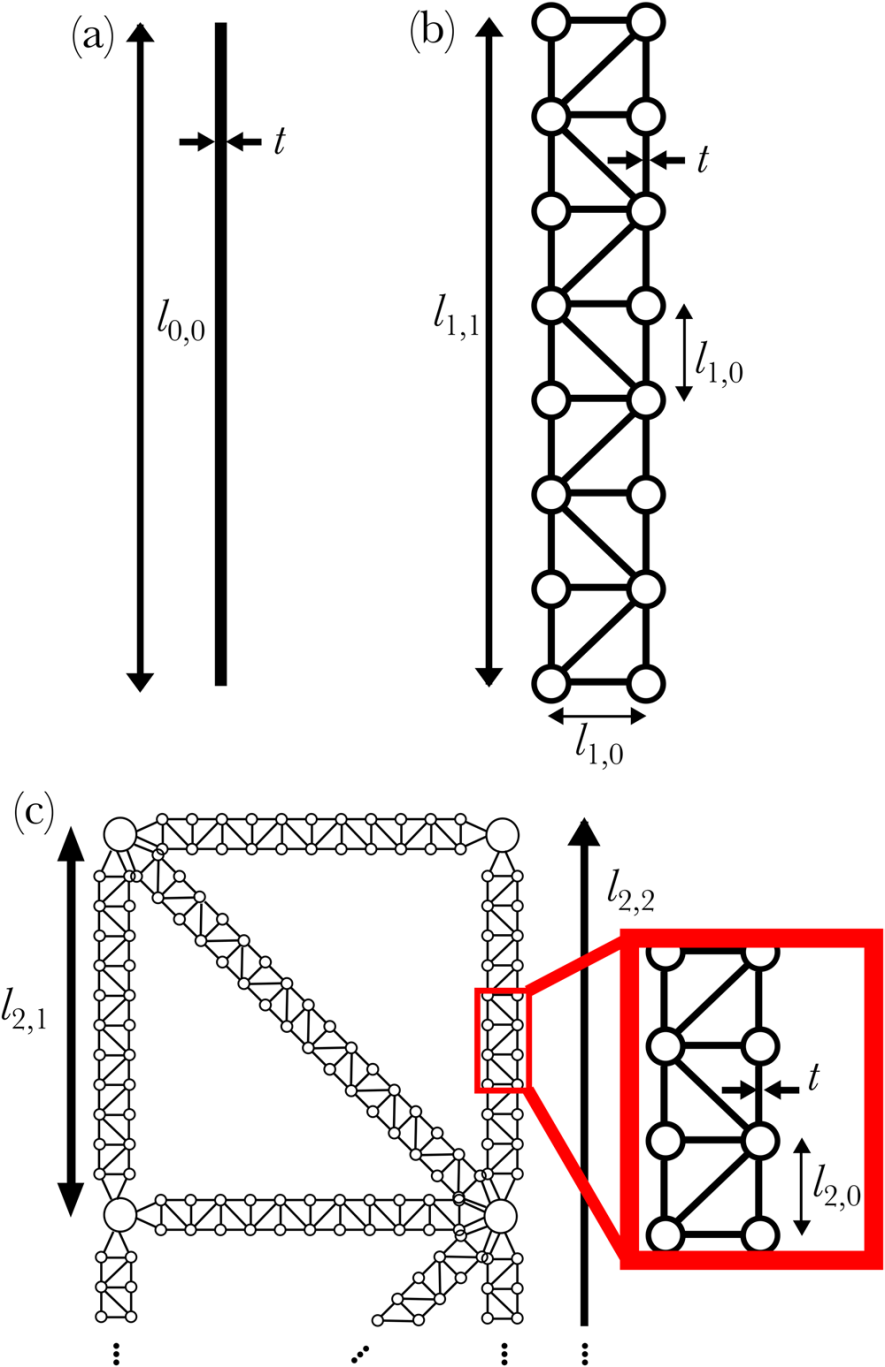
The progression from simple beam to hierarchical element, from left to right. Simple beam (**a**) is referred to as generation-0, a 2-d frame (**b**) is referred to as generation-1, a generation-2 structure (a frame made of frames) is shown in (**c**). The figure also introduces the notation parameterisng the frame structures.

Here we investigate a meta-material based on the re-entrant hexagonal lattice, a structure well known to have auxetic material properties^18,46,47^. We add to this lattice a substructure made up of hierarchical frames, where the degree of hierarchy can be varied allowing for systematic comparison between different designs. For reference, a structure with a single level of hierarchy (termed generation-0) will be investigated, this is the previously studied re-entrant lattice structure made up of simple beams. In the generation-1 structure, the simple beams in the generation-0 structure will be replaced with a 2-dimensional frame made up of simple beams (Fig. 1 shows an example lattice made up of generation-1 frames). In the generation-1 structure, *l*_1,1_ and *l*_1,0_ are the lengths of the frame and the constituent beams parallel to the long axis of the frame respectively, while *t* is the thickness of the beams (see Fig. 2(b)). All higher order geometries can be constructed in an iterative manner: taking a generation-*n* frame and replacing all simple beams with scaled generation-1 frames creates the generation-(*n* + 1) structure, Fig. 2 shows this procedure for generation-1 to -3. At all lengthscales with the exception of the longest, the lattice (or a coarse grained view of the lattice neglecting substructures smaller than *l*_*G*,*i*−1_) can be created using a Henneberg construction^48^, thus we ensure there are no mechanisms within the analogous freely hinged structure at these lengthscales^48^.

### Hierarchical lattice elements

In order to analyse the hierarchical meta-material, we must establish the mechanical properties of the two dimensional linkage element, we do this through the introduction of an “effective modulus”, “effective Poisson’s ratio” and an “effective thickness”; through these parameters we are able to encapsulate both the elastic response and elastic failure load of the linkage elements. In the case of the generation-1 design, analysing the equilibrium equations of the analogous freely hinged frame, we see that the stretching energy will be split between the two outer plates; thus the energy density due to stretching is simply,

2 $${U}_{S}=\frac{Yt}{(1-{\nu }^{2})}\,\int \,dx\,dy\,\{{e}_{11}^{2}\},$$

where *e*_11_ is the strain parallel to the neutral axis of the hierarchical structure. The energy density due to bending can be calculated as^49^:

3 $${U}_{B}=\frac{Yt{l}_{1,0}^{2}}{2(1-{\nu }^{2})}\,\int \,dx\,dy\,\{{(\frac{{\partial }^{2}w}{\partial {x}^{2}})}^{2}\}$$

where *w* is the out of plane deflection of the neutral axis of the frame. Through comparing these expressions with the standard expressions for the stretching and bending energy of a single plate^50^, it can be seen that the frame structure will behave equivalently to a simple plate with thickness $\tilde{t}$, Young’s modulus $\tilde{Y}$, and Poisson’s ratio $\tilde{\nu }$, where these parameters satisfy

4 $$\frac{\tilde{Y}}{1-{\tilde{\nu }}^{2}}=\frac{2t}{\sqrt{3}{l}_{1,0}}\frac{Y}{(1-{\nu }^{2})}$$

5 $$\tilde{t}=\sqrt{3}{l}_{1,0}$$

Through analysis of a single box section and considering the minimum energy for a given longitudinal displacement, it can be found that $\tilde{\nu }=0.5$. These values can be shown to agree with results presented in^49^.

### Iterative approach

One can generalise the above approach to any level of hierarchy. Here we utilise the notation of^49,51,52^, where the parameter *X* at hierarchical level *i* of a generation-*G* structure is referred to as *X*_*G*,*i*_ (*i* = 0 and *i* = *G* are the smallest and longest length-scale in the structure respectively). The number of box-sections used in the frames, *n*, is treated as a constant over all length-scales in this work, thus, we have the relationship

6 $${l}_{G,i}=n{l}_{G,i-1}\,\forall \,i\in [1,G-1].$$

The expressions for the effective properties of a generation-*G* structure at any hierarchical level must satisfy the following expressions^49^:

7 $$\frac{{\tilde{Y}}_{G,i}}{1-{\tilde{\nu }}_{G,i}^{2}}=\{\begin{array}{ll}\frac{2t}{\sqrt{3}{l}_{G,i-1}}\frac{Y}{(1-{\nu }^{2})} & {\rm{if}}\,i > 0\\ \frac{Y}{1-{\nu }^{2}}, & {\rm{if}}\,i=0\end{array}$$

8 $${\tilde{t}}_{G,i}=\{\begin{array}{ll}\sqrt{3}{l}_{G,i-1} & {\rm{if}}\,i > 0\\ t & {\rm{if}}\,i=0\end{array}$$

The effective Poisson’s ratio at all hierarchical levels is found to take the value ${\tilde{\nu }}_{G,i}=\tilde{\nu }$. These parameters can then be used in finite element simulations, placing beams with effective material and geometric properties in place of hierarchical frames. This methodology allows for the simulation of higher order structures where direct simulation would be intractable.

## Mechanical Properties of Meta-Material

Utilising the effective properties of the construction elements from the previous section, we are able to establish the mechanical properties of an auxetic meta-material of arbitrary hierarchical order. Furthermore, we derive scaling laws for the stiffness of the structure through analytic means. Finally we confirm the predictions of these two approaches through direct finite element simulations for the area of design space where such simulations are tractable. We first establish the stiffness of the lattice based meta-material before investigating the buckling response of the structures. COMSOL 5.3^53^ has been used for all finite element work presented here, assuming a linear constitutive law. Further detail regarding the implementation of the simulations can be found in the methods section.

### Stiffness

**Figure 3:**
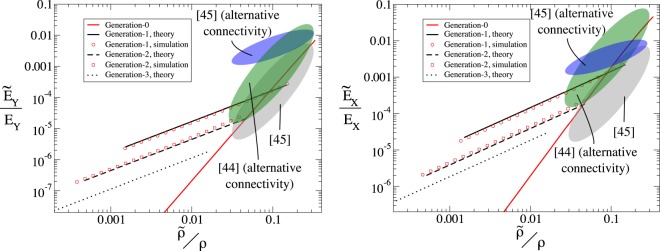
The scaling of relative stiffness vs relative density of re-entrant hexagonal lattice compressed in the *x* and *y*-directions for varying levels of hierarchy. The figure shows the stiffness increase in the transition to hierarchical designs in the limit of small densities. The shaded regions show the approximate values of relative stiffness vs relative density in previous works for other reentrant hexagon lattices (grey) and two lattices based on the reentrant hexagonal lattice with additional linkages (green and blue). It is notable that the increase in stiffness in references^44,45^ (green and blue shaded regions respectively) are accompanied by an increase in Poisson’s ratio (towards non-auxetic behaviour). Not only is the methodology presented here not associated by such an increase in Poisson’s ratio, but it also is generally applicable to any bending-dominated lattice. The hierarchical approach presented here also permits the creation of lattices with lower relative density for a given aspect ratio of component beams, reducing fabrication difficulties at low relative densities.

The stiffness of the auxetic frame with a given level of hierarchy can be established through finite element methods: A relative displacement is applied to the upper and lower boundaries for measurements of *E*_*Y*_ (or left and right boundaries for *E*_*x*_), where the orientation of the sample is as shown in Fig. 1; we measure the reaction force in the direction perpendicular to the imposed displacement. The frame geometry considered here is *n* = 10 for all frames, *L*_2_ = 20 mm, *L*_1_ = 10 mm and *θ* = 45°, the frames of *L*_*d*_ and *L*_2_ have beams of thickness *t* and *t*′ = *tL*_2_/*L*_*d*_ respectively; *t* is varied in order to vary the relative density of the meta-material. For hierarchical structures, computational expense is reduced by using a coarse grain view of the system utilising the effective material/geometric properties (see previous section). For generations-1 and 2 (for *n* = 10), these findings can be confirmed through direct simulation of the higher order structure. Figure 3 shows that the scaling law of Eq. (1) can be changed from *α* = 3 to *α* = 1 through the addition of hierarchy representing a substantial increase in stiffness in the lightweight regime.

The scaling law presented in Fig. 3 for both hierarchical and non-hierarchical elements can be derived following the approach of^33^. The deformation, *δ*, in a unit cell of a bending-dominated lattice, deforming due to in-plane bending, must follow^33,54^

9 $$\delta \sim \frac{F{L}^{3}}{D},$$

where *F* is an external force per unit length on a lattice with lattice elements of characteristic length *L*, and *D* is the flexural rigidity of the plate structure. For a 2-d plate structure, the flexural rigidity will be calculated as^50,54^,

10 $$D=\frac{Y{t}^{3}}{12(1-{\nu }^{2})}$$

For a 2-d lattice, the stress, *σ*, acting on a unit cell is proportional to *F*/*L*, while the strain, *ε*, will scale as *δ*/*L*. The density of the lattice is given by,

11 $$\tilde{\rho }\sim \frac{t}{L}\rho .$$

Thus, in a lattice made up of simple beams, combining the above observations with Eqs (9, 10 and 11), we see that the appropriate scaling in Eq. (1) is given by *α* = 3.

When we consider a generation-1 frame, as shown in Fig. 2 (middle), noting *L* ≈ *l*_1,1_ and using Eqs (6, 9 and 10), with the use of the effective beam properties from Eqs (7 and 8), we find

12 $$\sigma \sim \frac{t}{L}{n}^{-2}Y\varepsilon .$$

Noting that the density of the lattice still scales as Eq. (11), we see that for constant *n*, we obtain a new scaling from Eq. (1) of *α* = 1. This beneficial scaling relationship, *α* = 1, can also be derived for higher order hierarchical frames.

### Poisson’s ratio

**Figure 4:**
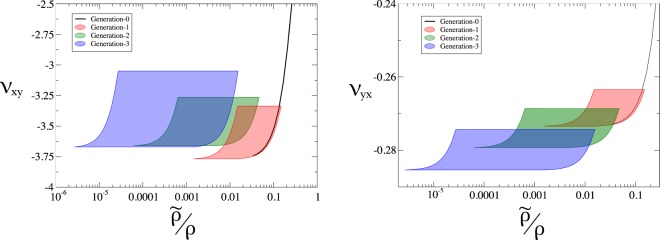
The range of possible Poisson’s ratio for a structure of a given relative density. With increasing hierarchical order of the structure, a broader range of Poisson’s ratio is realisable for a fixed relative density.

The Poisson’s ratio of this geometry can be approximated through a freely hinged assumption^47,55^. This model is dependent only on the lattice parameters at the largest length-scale (*L*_1_, *L*_2_, *θ* in Fig. 2) and the magnitude of strain applied. Deviations from the predictions of this idealised model will depend on the comparative energetic cost of stretching vs bending of the lattice elements; thus these deviations will depend only on the (effective) aspect ratio of the construction elements. Thus, for a given relative density of generation-0 structure (assuming both elements are of equal aspect ratio), the Poisson’s ratio will be fixed. The hierarchical structure gives an additional freedom, however; we are able to vary the relative density of the lattice, without changing the effective aspect ratio construction element (${\tilde{t}}_{G,G}/{l}_{G,G}$) through varying the geometry at smaller length-scales. This gives us an additional freedom to set the Poisson’s ratio of the material (by setting *L*_1_, *L*_2_, *θ* and ${\tilde{t}}_{G,G}/{l}_{G,G}$) and vary the relative density of the lattice (through altering the structural parameters at the smaller length-scales). We show the region of design space (${\nu }_{xy},\tilde{\rho }/\rho$) that is realisable for a lattice with 0, 1, 2, and 3 levels of substructure in Fig. 4, this plot is for a lattice with *Y* = 50 MPa, *L*_1_ = 10 mm, *L*_2_ = 20 mm and *θ* = 45°, subject to the restriction that all aspect ratios in the structure are between 10 and 100.

### Strength

**Figure 5:**
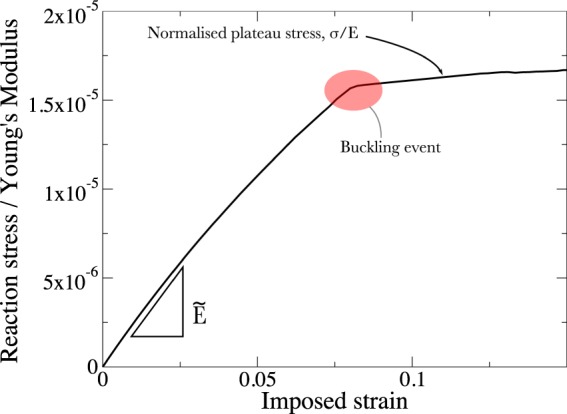
A typical non-dimensionalised stress strain curve for the lattices under investigation here. A plateau follows the buckling event.

Finally we investigate the buckling strength of the auxetic lattice. As described in^33^, the typical stress strain curve is described by a roughly linear relationship until failure when a plateau of stress with increasing strain is observed, this relationship is shown in Fig. 5. For lightweight lattices, high aspect ratios are advantageous, and in this limit elastic instability is likely to be the active mode of failure^52^. For a frame in 2-d of length *L* and flexural rigidity *D*, subject to compressive loading per unit length *F*, elastic instability will occur when

13 $$F=\frac{\pi D}{{L}^{2}}.$$

Here, we take a lattice designed such that both the horizontal and diagonal elements of Fig. 1 have the same buckling load. For a given relative density of frame, the structural elements that make up the lattice can be optimised on all component length-scales. In order to do this, we follow the methodology of^49^: the parameters *t* and {*l*_*G*,*i*_} are set such that the frame is on the point of elastic failure on all length-scales simultaneously. Utilising the expressions (7, 8, 10 and 13), it can be shown that the buckling load of a single hierarchical frame will scale as

14 $$\frac{F}{{Y}_{m}L}\sim {(\frac{\tilde{\rho }}{\rho })}^{\frac{G+3}{G+1}}\mathrm{.}$$

**Figure 6:**
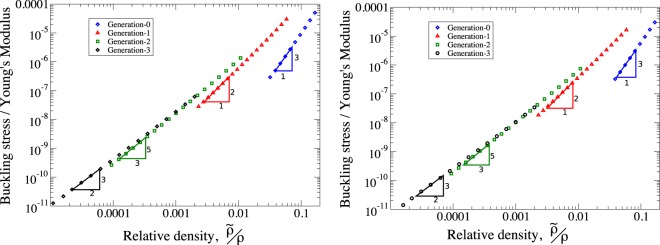
The scaling of buckling load vs relative density of bow-tie lattice compressed along the *x* and *y*-directions for varying levels of hierarchy. Figure shows the increase in buckling resistance for increasing generation of structure for lightweight structures. The points shown are results of finite element simulations using effective beam properties where all elements in the hierarchical structures have aspect ratio between 20 and 100.

Given *σ* ~ *F*/*L*, it is expected that the normalised stress at which the lattice will exhibit elastic instability (*σ*/*Y*_*m*_) will follow the same scaling with relative density as shown in Eq. (14) ^52^. Direct simulation of the geometries investigated here is not possible as the ratios *l*_*G*,*i*_/*l*_*G*,*i*−1_ increases quickly with smaller optimisation load and thus the number of elements quickly becomes intractable. To circumvent these difficulties, Eqs (7 and 8) are used and finite element simulations are performed using the effective beam properties, as in the previous section. The scaling in Eq. (14) is observed from the output of finite element simulations as shown in Fig. 6. Increasingly good agreement between scaling law prediction (Eq. (14)) and simulation are found for increasing aspect ratio.

## Summary

In this paper lightweight auxetic lattices with unprecedented stiffness have been designed through the use of hierarchical substructures. The structural response of these hierarchical architectures has been analysed through a combination of computational and analytic methodologies. Using the results of an analytic work, coarse grain simulations have allowed the analysis of structural systems where direct computation would prove intractable. Scaling arguments relating both stiffness and strength to weight ratios have been established, and are found to be in agreement with computational work. Using a scaling law argument, we have shown that through the use of hierarchy, enhanced stiffness and strength can be achieved in a wide class of bending-dominated lattices.

The mechanical performance of these lattice based structures when subject to imperfection is an open, and important, question. Though initial studies have shown that the hierarchical element used here subject to single beam imperfections is not altered by increasing hierarchy^49^, it is not known how manufacturing tolerances will affect the performance of the lattices presented here. It is notable, however, that hierarchy is often found in naturally occuring structures that are renowned for their insensitivity to imperfections^4,37–40^, this leads to the tantalising possibility of combining augmented strength and stiffness together with high tolerance to imperfections.

While all structures presented here with one or more level of hierarchy have a linear relationship between relative stiffness and relative density, the maximum stiffness is achieved for generation-1 structures. With increasing generation, the scaling relationship between buckling stress and relative density is manipulated in a beneficial manner, however. It is therefore suggested here that in situations where strength is of critical importance and increased stiffness desirable, higher generation structures may still be beneficial. It is also of interest to note that while increasing the generation leads to decrease in stiffness (from generation-1 and higher), these higher order structures are still stiffer than their generation-0 counterpart in the lightweight regime. Finally it is noted that the hierarchical designs presented here allow for structures of lower relative density to be fabricated while using elements of relatively low aspect ratio.

This work has potential application in the design of stiff, strong auxetic meta-materials where it has been observed that auxetic behaviour in conventional lattices is not compatible with stretching dominated deformation modes which imply high stiffness^35^. We have shown that through structural design on multiple length-scales, one can achieve auxetic meta-material properties while retaining stiffness comparable with stretching-dominated architectures in the limit of low relative density.

The significance of this work to a wide variety of technological application is enhanced by the recent fabrication of mechanical meta-materials on micro/nano scale through use of nanomembrane technologies^9^ and digital printing techniques^55–57^. The use of nanomembrane technologies restricts the fabrication such lattices to plate-like structures that, under compression, will experience out of plane elastic instabilities; such structures could be artificially restrained to remain planar, in which case all the results of the present work will hold, or under tension, without restraint, the lattice is expected to follow the results obtained in section 2.1. The practical application of the architectures presented here can no doubt be increased through the use of graphene^58–60^, poly(phenylacetylene)^61^ or other 2-d nano-layer based materials: such structures represent a possible route to creating 2-d lattice based auxetics on the nano/micro length-scale with multiple length-scales present within the structure.

## Methods

All finite element work here has been undertaken using COMSOL 5.3. In all cases mesh refinement studies were performed to ensure convergence of the results.

The stiffness of the meta-materials was established (Fig. 3) using the 2-d structural mechanics module utlising Euler-Bernoulli beam elements. Stationary studies were undertaken on a lattice *N*_*x*_ = 4, *N*_*y*_ = 5, *Y*_*m*_ = 50 MPa, *θ* = 45°, *L*_1_ = 0.01 m and *L*_2_ = 0.04 m, while *t* was varied in order to vary the relative density of the frame. Both the horizontal and diagonal frames in Fig. 2 were made up of *n* = 10 box-sections. For investigations on the stiffness, *E*_*Y*_ (*E*_*X*_), vertical (horizontal) displacements were imposed on the upper and lower (left and right hand side) boundaries creating a compressive strain, the boundaries were permitted to translate in the *x* (*y*) direction but not rotate, a single boundary was fixed in order to restrain translation of the whole structure. The reaction forces were measured on the lower (left hand side) boundaries in order to establish the stiffness of the meta-material. The boundaries parallel to the displacement on the extreme edges of the sample (right and left for *E*_*Y*_ studies, upper and lower for *E*_*X*_) were restrained such that no rotation was permitted, however translation was allowed. Studies with greater *N*_*x*_ and *N*_*y*_ were undertaken utilising effective beam properties, the same stiffness as is presented in Fig. 3 was found.

The stress-strain curve shown in Fig. 5 was obtained through a stationary study in 2-d using the solid mechanics of COMSOL 5.3. Imperfections were added to the lattice in the form of the first five eigenmodes obtained through linear study. These imperfections were added to the lattice through the use of MeshPerturb 1.0^62^. The lattice parameters for this study were *N*_*x*_ = 15, *N*_*y*_ = 15, *L*_1_ = 0.01 m, *L*_2_ = 0.04 m, *t* = 1 mm, *θ* = 45°.

Investigations into the buckling response of the structure were undertaken using the solid mechanics module of COMSOL 5.3. The lattice parameters (*N*_*x*_, *N*_*y*_, *L*_1_, *L*_2_, *θ*) were the same as in the stiffness studies. Only the effective beam properties method was used in order to make the simulations less computationally expensive as for optimised frames *l*_*G*,*i*_/*l*_*G*,*i*−1_ increases quickly creating structures that can’t be simulated directly. The lattice elements were optimised for buckling load such that both the horizontal and vertical elements (see Fig. 1) were optimised for the same value of *f* ≡ *F*/*Y*_*m*_*L* using the method presented in^49^. The value of *n* and *t* were allowed to vary in order to create optimal frame elements for a given *f*, and the buckling load of the lattice was then obtained through a linear buckling study.

## Other Geometries

**Figure 7:**
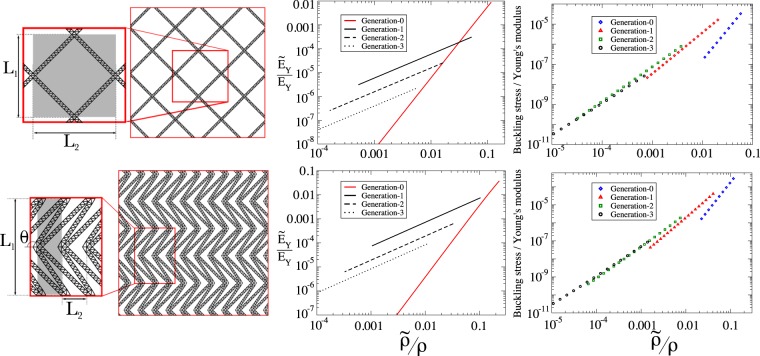
Two further geometries investigated here: a rhombic lattice and a double arrow lattice (left, upper and lower repsectively). Also shown are unit cells (grey shadow) and the notation used in the parameterisation of the lattice. Middle shows the relative stiffness in the *y* direction for the generation-0 structure (red) and structures with hierarchy (generation-1 to -3). As predicted in section 2.1 the relationship between relative stiffness and relative density is linear for the higher order structures, while for the generation-0 structure, the power in Eq. (3) is *α* = 3. The relative strength of the two architectures is shown on the right hand side. In both cases, the scaling of the stress required to induce buckling follows the prediction of Eq. (14).

To demonstrate the wide applicability of the scaling laws found in sections 2.1 and 2.3, here we use the hierarchical design methodology to enhance the stiffness and strength of two further architectures. Here, we take two further bending-dominated lattices: a rhombic lattice and double arrow lattice as examples of non-auxetic and auxetic^63^ geometries respectively. These structures are shown in Fig. 7 (left) where the notation is also introduced. The rhombic lattice has a unit cell of dimensions ${L}_{1}=1,\,{L}_{2}=\sqrt{3}$ cm, while the double arrow unit cell is parameterised by the values *L*_1_ = 1, *L*_2_ = 0.5 cm and *θ* = *π*/6 rad. For the stiffness results, the value of *n* is held fixed at *n* = 10 resulting in a linear relationship between relative density and stiffness for structures with hierarchical substructures, as predicted above (Fig. 7 middle); for the investigation into strength, the hierarchical elements are optimised for compressive strength following the procedure of ref.^49^. In Fig. 7 right, the buckling stress is plotted against relative density for different generations of structure. It is found that the scaling predictions given in Eq. (14) are obtained for all the geometries investigated here.

## Data Availability

There is no experimental data in this paper.
